# COVID‐19 lockdown measures impacted citizen science hedgehog observation numbers in Bavaria, Germany

**DOI:** 10.1002/ece3.8989

**Published:** 2022-06-17

**Authors:** Fabio S. T. Sweet, Thomas Rödl, Wolfgang W. Weisser

**Affiliations:** ^1^ Terrestrial Ecology Research Group Department of Life Science Systems School of Life Sciences Technical University of Munich Freising Germany; ^2^ Landesbund für Vogelschutz e.V Hilpoltstein Germany

**Keywords:** Bavaria, citizen science, coronavirus, hedgehog, lockdown

## Abstract

The COVID‐19 pandemic has led to temporary changes in human–animal interactions due to changes in human activities. Here, we report on a surge in hedgehog observations during the first COVID‐19 lockdown in Germany in 2020, on the citizen science Web portal “Igel in Bayern” (Hedgehogs in Bavaria) in Germany. This increase in comparison with previous years was attributed to an increase in the number of people reporting hedgehog observations, rather than an increase in the number of hedgehog observations made by each observer. Additionally, in contrast to other studies on the effects of a COVID‐19 lockdown on observations recorded by citizen science projects, the share of observations made in more urbanized areas during the lockdown time was not higher than the change observed in less urbanized areas. This is possibly a result of the differences in COVID‐19 measures between Germany and other countries where preceding studies were carried out, in particular the lack of measures limiting traveling outdoor activities for citizens.

## Graphical abstract

A significant increase in Hedgehog observations and people observing hedgehogs can be observed in the first COVID‐19 lockdown in Bavaria, Germany, based on data from the NGO "Landesbund für Vogelschutz." The increase in observations was attributed to the increase in observers. In contrast to other studies on the effect of a COVID‐19 lockdown on observations recorded by citizen science projects, the share of observations made in more urbanized areas was not higher than the change observed in less urbanized areas.

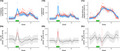

## 1 INTRODUCTION

At the onset of the COVID‐19 pandemic, many countries issued lockdowns to reduce the number of contacts between people, as a measure to limit the spread of the virus. In several countries, these lockdowns have increased public interest in urban nature (Roll et al., 2021). Analyses of citizen science platforms have shown that lockdowns can result in an increase (Basile et al., 2021; Crimmins et al., 2021; Manenti et al., 2020), but also in a decrease (Crimmins et al., 2021; Kishimoto & Kobori, 2021; Rose et al., 2020; Sánchez‐Clavijo et al., 2021) in animal observations by citizen scientists compared with preceding years. Further analyses showed that increases were largely due to increases in observations in urban areas, while no change, or even a decrease in the number of observations, was reported from nonurban areas (Basile et al., 2021; Sánchez‐Clavijo et al., 2021). Other studies have reported various positive effects of lockdowns on urban wildlife (Driessen, 2021; Manenti et al., 2020), but more detailed investigations, for example, on birds, suggested that animal responses were largely behavioral, that is, animals were more present in areas with less traffic and fewer humans, but there was no lasting increase in population density (Gordo et al., 2021). There are also reports of negative effects on wildlife originating from changes in human behavior during the COVID‐19 pandemic (Gilby et al., 2021; Hiemstra et al., 2021).

In Germany, the first major COVID‐19 lockdown lasted from March 22, 2020, to May 4, 2020 (weeks 13–19), and entailed restrictions on movement and contacts among people (Bayerische Staatskanzlei, 2020). Many recreational amenities were closed during the lockdown, but people were allowed to do outdoor activities, either alone or with another person from their own household, and there were no restrictions on where people could go to within the country.

Here, we report on a COVID‐19 effect on observations of hedgehogs made through a citizen science portal in Germany. The NGO "Landesbund für Vogelschutz" (LBV) in Bavaria uses an app and a Web portal to monitor the occurrence of hedgehogs in Bavaria (“Igel in Bayern,” www.igel‐in‐bayern.de). Observations started in 2015. We use data collected by the portal to ask the following questions:

1. Did the first major lockdown in Germany lead to an increase in the number of hedgehog observations on the LBV app and portal?
2. Is a change in the number of observations due to an increased number of people making observations, or to an increased number of observations made by each observer?
3. Are there differences between urban and nonurban areas in any changes in the number of observations?

## 2 METHODS

Reporting of hedgehogs in the LBV app can be made with or without registering as a user. Observations of people that did not register contained the same data‐fields as those of people that did register, except for the observer‐ID. We obtained 107,440 observations of hedgehogs from the LBV database. Duplicate observations, as well as observations made outside of Bavaria, were eliminated from the dataset (Appendix S1). The year 2015 was also omitted from the analysis because it was the first year of the citizen science project and the year was not complete. After cleaning, 83,008 observations remained that were summarized per week. The years 2016–2019 were grouped together so that the 14,261 observations made in 2020 could be compared with expected values based on the preceding 4 years. To compare the number of hedgehog observations in 2020 with those of 2016–2019, we used generalized additive models and a spline smoothing function for the weekly observations (Appendix S1). Confidence intervals were used for the comparison between years. The same analysis was made to compare the weekly numbers of registered users that did observations in 2020 with those of 2016–2019.

To analyze the mean number of observations made by a person each week and compare this between years, we divided the number of observations made by registered users per week by the number of registered users in the same week and used these numbers in our analysis. Generalized additive models constructed conducted with the “mgcv” (Wood, 2021) package, and outputs were compared with “itsadug” (Van Rij et al., 2020) package in R version 4.0.2 (R Development Core Team, 2008).

To analyze whether changes in the number of observations in 2020 differed between more and less urbanized areas, we drew a 200 m radius around every hedgehog observation and calculated the mean imperviousness density, using the 20 m × 20 m resolution 2015 impervious surface density map of the European Union's Copernicus Land Monitoring Service (Langanke, 2016) as a measure of urbanization. The 200 m radius was chosen so that the context of the broader environment in which the observations were made would not be lost, so small islands of imperviousness would not result in small green inner‐city area being classified as less urbanized. Observations were then divided into increments of 20% impervious surface density. The percentage of observations and observers during the weeks of the first COVID‐19 lockdown in every imperviousness class was compared across the years of interest as follows: similar to Crimmins et al. (2021), linear models were built between 2016 and 2019 for every imperviousness class and these were used to create an expected value with a 95% prediction interval for 2020 (Appendix S1). These were used as a measure of significance by comparing the realized value of 2020 to the prediction interval.

## 3 RESULTS

### 3.1 Increase in hedgehog sightings during the COVID‐19 lockdown

**FIGURE 1:**
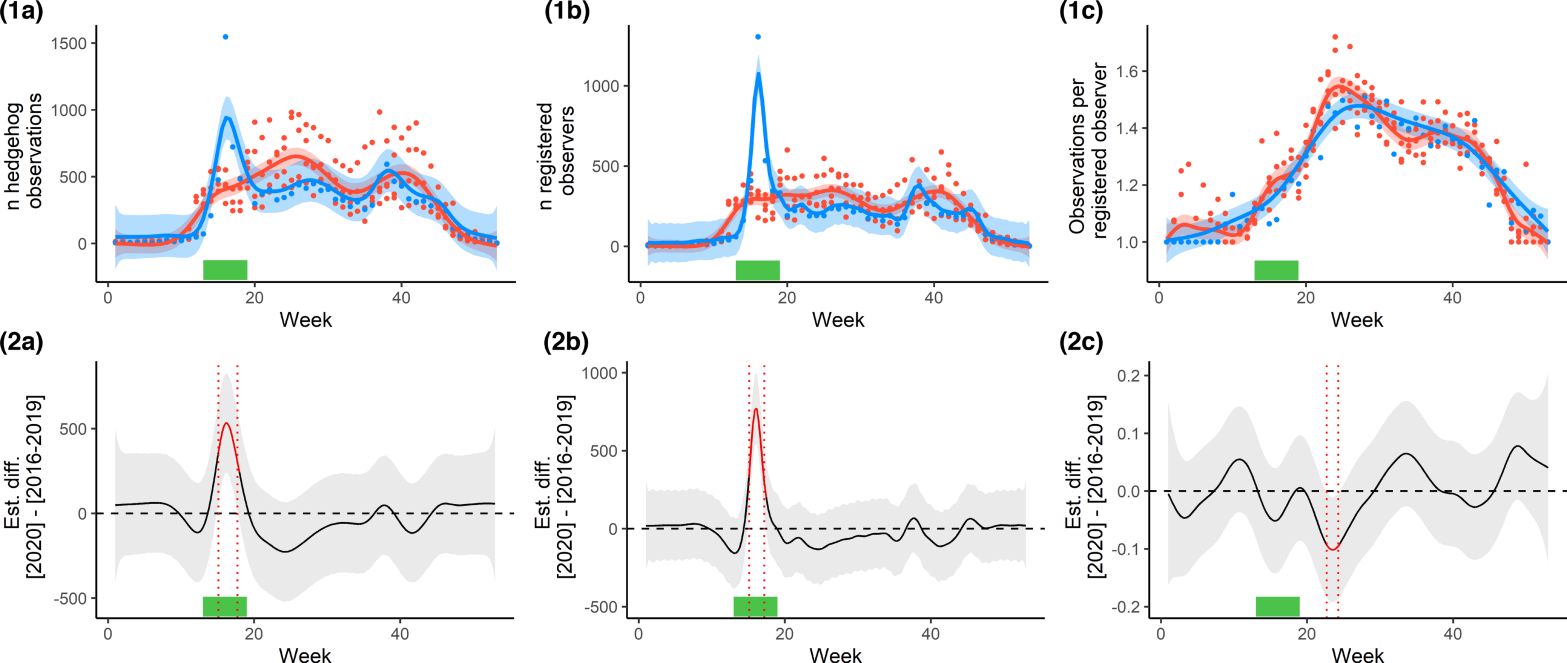
Differences in hedgehog observations in Bavaria, Germany, reported on the LBV’s “Hedgehogs in Bavaria” Web platform, between 2020, that is, the year of the first COVID lockdown, and pooled data of 2016–2019. (a) Weekly absolute number of hedgehog observations. (b) Weekly numbers of registered users that reported hedgehog observations. (c) Weekly numbers of hedgehog observations per registered user. (1) (top row of figures) Raw data and temporal trends, fitted using spline functions, separately for 2020 and 2016–2019. (2) (bottom row of figures) Difference between the 2020 and 2016–2019 estimates of (1). The lockdown period is indicated by a green bar; in 1, the red line indicates 2016–2019 and the blue line indicates 2020, and in 2, the red time period indicates significant differences between 2020 and 2016–2019

In 2020, there was a peak in the number of observations between the start of week 15 [06.04.2020–12.04.2020] and the end of week 17 [20.04.2020–26.04.2020] (Figure 1: 1.A and 2.A), that is, during the period of the lockdown. No such peak was observed in the years 2016–2019, and the confidence intervals indicated that the difference was statistically significant. In the 16th week of 2020, 1547 sightings were recorded into the LBV’s platform; this is a more than threefold increase compared with the average of 430 observations in that week based on the four preceding years. The model scored an *R* ^2^ of .72.

### 3.2 Increase in the number of registered users during the COVID‐19 lockdown

The number of registered users in 2020 was higher than the number of registered users in 2016–2019, from the start of week 15 to the start of week 17 (Figure 1: 1.B and 2.B). In the 16th week of 2020, 1305 registered users recorded hedgehog sightings on the LBV platform; this is a more than fourfold increase compared with the average of 287 registered users in the four preceding years. The model scored an *R* ^2^ of .78.

### 3.3 No change in the number of observations per registered user

The model indicated no significant change in the number of hedgehog observations per registered user during the first COVID‐19 lockdown. There was, however, a minor significant dip in the number of hedgehog observations per registered user several weeks after the lockdown, from the end of week 22 [25.05.2020–31.05–2020] to the start of week 24 [08.06.2020–14.08.2020] (Figure 1: 1.C and 2.C). The model scored an *R* ^2^ of .88.

### 3.4 No significant changes in the percentage of observations across different levels of urbanization

**FIGURE 2:**
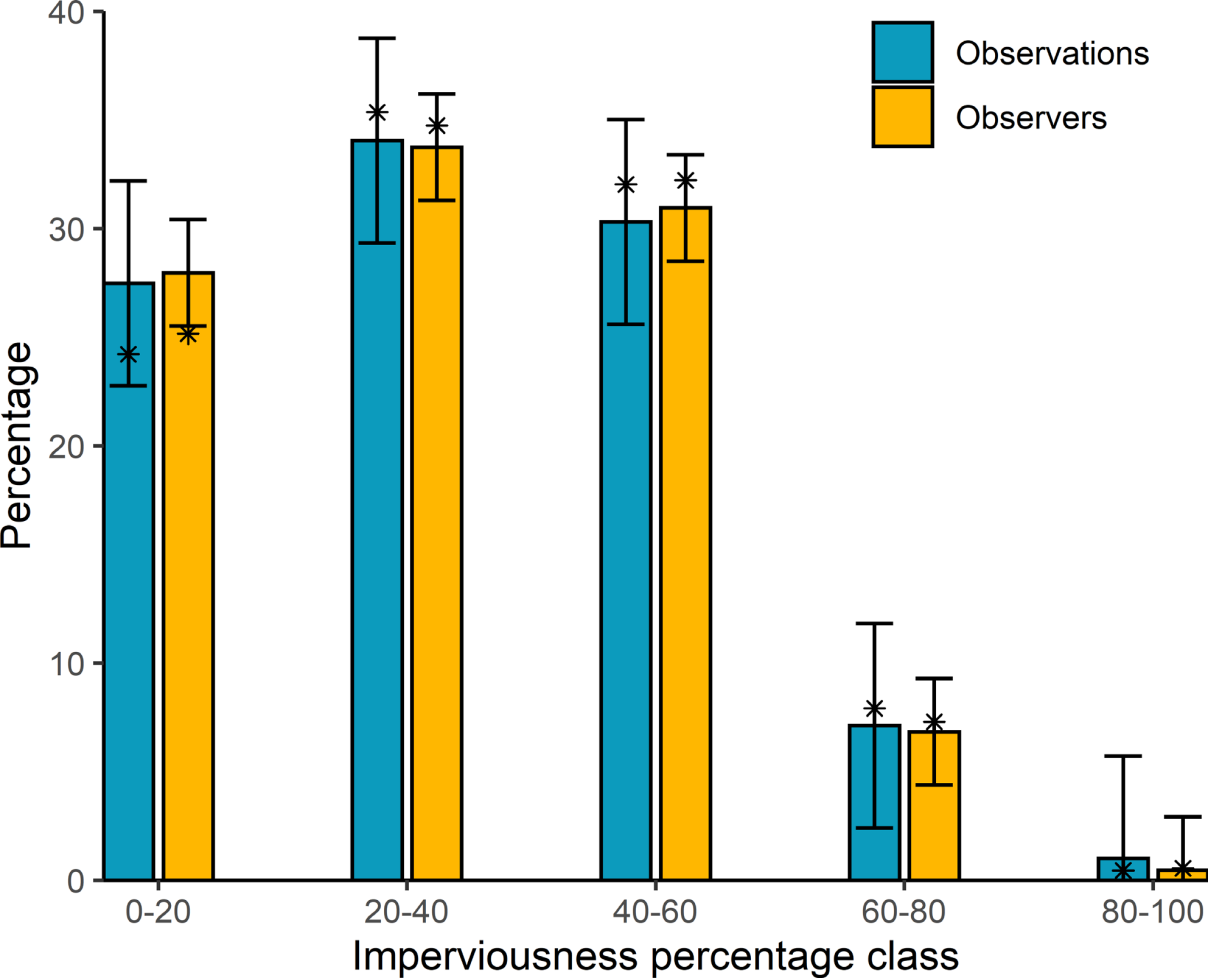
Predicted (bars) and realized (*) percentages of observations (blue, left) and observers (orange, right) per imperviousness class, for weeks 13–19 in 2020. Bars indicate predicted values for 2020, based on observations from the years 2016–2019 in the same period for each class of imperviousness. Error bars indicate 95% prediction intervals of the predicted values of linear models (Appendix S1). Stars* indicate realized values in 2020. Realized values falling outside of their respective modeled prediction's 95% prediction interval are treated as significantly different from expectations (significant deviation was only found for observers in the 0–20 imperviousness class). The full model output, including values for the preceding years, can be found in Appendix S1, Figures A1 and A2

No significant differences were registered between the realized and predicted percentage of observations in different levels of urbanization in 2020. There was, however, a small but significant difference in the percentage of observers that did observations at the lowest level of imperviousness, compared to what would be expected from the prediction based on preceding years. That value was 25.16%, 2.81% less than the predicted value of 27.97% (Figure 2; Appendix S1).

## 4 DISCUSSION

We found a statistically significant increase in the number of hedgehog observations during the first COVID‐19 lockdown period 2020 compared with the same time‐period of preceding years. This result mirrors results of other studies that investigated the effect of the COVID‐19 lockdown on animal observations made in citizen science projects (Basile et al., 2021; Crimmins et al., 2021; Manenti et al., 2020). Additionally, we found a pattern that to our knowledge was not reported before, namely that this increase was primarily due to an increase in the number of observers, and not an increase in the number of observations per observer. In contrast to other studies, we found that the increases in both the number of observations and the number of observers during the lockdown period occurred equally in both more urbanized and less urbanized areas.

An increase in the number of hedgehog observation can be due to a higher observation activity of humans, or a higher activity of hedgehogs. It is very likely that the increase in hedgehog observations during the first German COVID‐19 lockdown is attributable to the increase in the number of human observers, and not to an increase in hedgehog numbers or hedgehog activity. This is corroborated by the finding that there was no difference in the mean number of hedgehogs that participants reported during the lockdown period vs. the preceding years (Figure 1: 1.C and 2.C).

After the lockdown period, the number of observations and observers quickly returned to normal. There was even a short significant decrease in the mean number of hedgehogs participants reported a few weeks after the lockdown compared with the preceding years. It can be argued that this is due to a loss of interest in looking for hedgehogs because other temporarily unavailable potentially competing activities had resumed. Competing activities could be, for example, activities pertaining the hospitality industry and cultural activities such as visiting museums, activities which were not possible during the first COVID‐19 lockdown in Germany.

While other studies have found that there was an increase in the share of urban observations as a total of all observations made during the lockdown, we did not find such a difference between more and less urbanized areas. A possible reason for the differences in the share of observations in more versus less urbanized areas in our study compared to others (Basile et al., 2021; Crimmins et al., 2021; Kishimoto & Kobori, 2021; Manenti et al., 2020; Rose et al., 2020; Sánchez‐Clavijo et al., 2021) is the differences in measures between Germany and the countries investigated in other studies (Hirsch, 2020). During the first lockdown in Germany, many recreational amenities were closed. While people were allowed to do outdoor activities, either alone or with another person from their own household, there were no restrictions on where people could go to within the country. This is different from, for example, France and Italy, where inhabitants were only allowed to go outside alone and near their home, or the United Kingdom, where nonessential movements were banned and inhabitants were only allowed to go outside once per day, alone or with people from the same household (Hirsch, 2020). A report by the German hiking institute (Deutsches Wanderinstitut) indicated that people in Germany went on hikes more often than normally in April and May of 2020, and those responsible for the hiking routes indicated that there were often more hikers on the hiking routes (Smolka et al., 2021). Nonetheless, there was a decrease in physical activity of people, as shown, for example, for the German state Bavaria (Huber et al., 2020). We do not know where the observers live and how far they travelled to the observation point, as people did not register with their address on the portal. It may be that more observations were made near their house, or that they went further away. We cannot distinguish between a situation in which more people left the house during the lockdown and a situation in which the same number of people went outside during the lockdown but a higher fraction of them reported hedgehogs. In both cases, the number of observers and the number of observations would increase, and we think that the first scenario is more likely. The difference in restrictions and subsequent response in activity patterns between Germany and the countries investigated in other studies could nonetheless explain why there was, in contrast to studies in other countries, no noticeable increase in the share of hedgehog observations in more urbanized areas, compared with less urbanized areas in our study while the absolute number of observations increased.

## 5 CONCLUSIONS

Our study showed that the first COVID‐19 lockdown in Germany led to an increased number of people reporting hedgehog sightings on a citizen science portal, resulting in a higher number of hedgehog observations in this period. When the lockdown was over, the number of observers dropped back, so that there was not significant difference anymore to preceding years. Our results therefore suggest that there is great potential to increase animal observations at times when people have time for such activities, but also that reporting animal observations to a citizen science portal is limited by the presence of other potential activities. Our results also suggest that the way in which human movement patterns were restricted during the first COVID‐19 lockdown influenced the reporting of animal observations in citizen science portals. In the case of Germany, humans were not confined to stay in areas with higher human population density, and hence, reporting of hedgehogs also occurred in areas that were less urbanized where people could go for outdoor activities. Thus, considering the circumstances under which citizen science data were collected can help to interpret observed changes in reporting patterns.

## AUTHOR CONTRIBUTIONS

**Fabio S T Sweet:** Conceptualization (lead); Data curation (equal); Formal analysis (lead); Investigation (lead); Methodology (lead); Project administration (equal); Software (lead); Visualization (lead); Writing – original draft (lead); Writing – review & editing (equal). **Thomas Rödl:** Data curation (equal); Resources (equal); Writing – review & editing (equal). **Wolfgang Weisser:** Conceptualization (equal); Formal analysis (supporting); Funding acquisition (lead); Methodology (supporting); Resources (lead); Supervision (equal); Writing – review & editing (equal).

## CONFLICT OF INTEREST

The authors declare no conflict of interest.

## Supporting information

AppendixS1

## Figures and Tables

**FIGURE A1:**
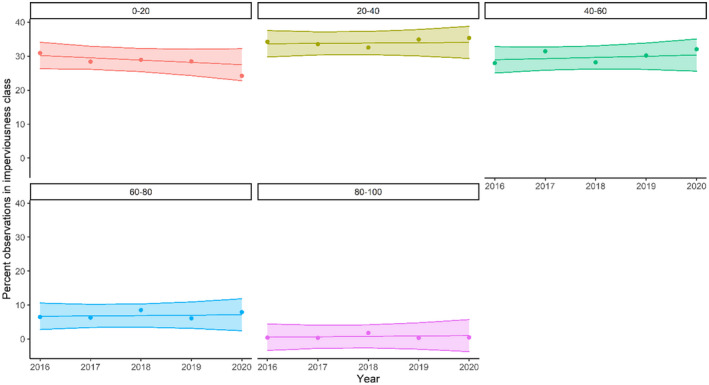
Percentage of observations within urbanization classes from 2016–2019, extrapolated into 2020. Each panel shows the results for one imperviousness classes. The dots indicate the yearly realized percentage of observation in each imperviousness class each year. In none of the imperviousness classes, the realized value of 2020 significantly differed from extrapolated expectations. *Y*‐axis: The percent of observations in each imperviousness class. *X*‐axis: Year. Plot facets and colors: Imperviousness classes. Line ± CI: Linear model per imperviousness class based on 2016–2019, extrapolated towards 2020

**FIGURE A2:**
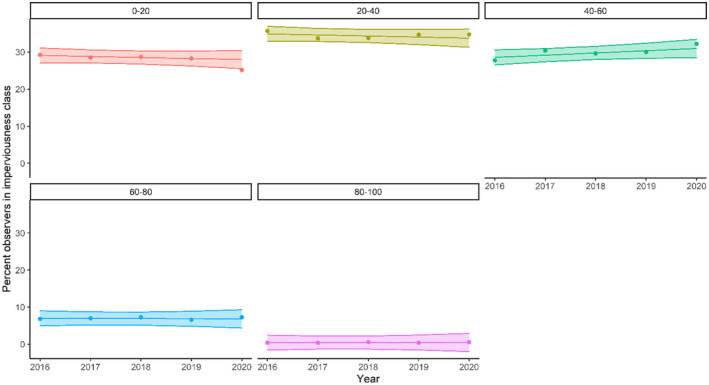
Percentage of participants (observers) within urbanization class from 2019–2019, extrapolated into 2020. Each panel shows the results for one imperviousness classes. The dots indicate the yearly realized percentage of observers in each imperviousness class each year. There is a very mild deviation in realized value from expected value in the lowest imperviousness class. No significant deviations in the other imperviousness classes. *Y*‐axis: The percent of observers in each imperviousness class. *X*‐axis: Year. Plot facets and colors: Imperviousness classes. Line ± CI: Linear model per imperviousness class based on 2016–2019, extrapolated towards 2020

## Data Availability

Code necessary to reproduce the analysis is available in the supplementary material. Data required to reproduce the analysis is available on Dryad via https://doi.org/10.5061/dryad.gmsbcc2qp. For the complete dataset with additional variables, please contact the Landesbund für Vogelschutz e.V., Bavaria.

## APPENDIX 1

### A Duplicate observations

Because observations could be made by both registered and non‐registered participants, of which only the observations of registered participants could be connected to an anonymized participant ID, while the non‐registered participants by default got a Participant ID of “0,” these two groups had to be cleaned individually.

For preparation, the data were split into two documents: one with registered participants and one with non‐registered participants. After removal of duplicates, the final datasets were then again merged. Here, we describe the steps to remove duplicate observations, followed by a reproducible ArcGIS‐specific description.

First, a shapefile (1) with 50m buffers around each datapoint was created. The shapefile with these buffers was then split into multiple shapefiles (2): one for each day in the dataset.

For each of these shapefiles, that is, for every day of the calendar day where there were observations, the observation points were pairwise dissolved, so that two observations where the buffers overlapped to any extend and that had the same data on them would be considered as identical observations. If the observations were considered to be identical, that is, referring to the same hedgehog seen by the same person, the two observation buffers would be merged, creating a larger buffer made from the combined dissolved buffers. These new buffers would gain the observation‐ID of the first if the observations out of which the dissolved buffers were made.

After this was made for each created shapefile created in (2), that is, for each day, these shapefiles (2) were merged to create a single shapefile with all the new dissolved buffers in it (3). This new shapefile was then spatially joined (overlapped) with the original point‐data, and only points that had the same data as a buffer (from 3) that overlapped them were kept. This gave the data three different ID fields: (a) JOIN_FID’s, that is, new ID’s made for points that were overlapped with a certain buffer; (b) FIRST_id's, which were the ID’s of the first observation that was part of any combined buffer, and (c) the original observation‐ID. These selected points were exported into a new shapefile, which was then exported into a CSV file. In Excel, the duplicate JOIN_FID’s would then be marked, and those that did not have the same FIRST_id field as the original file's observation‐ID would be removed. This was made for both the data with registered participants and with unregistered participants, albeit with some minor differences, as follows:

#### Detailed ArcGIS procedure to remove duplicate observations among registered participants

Here, we describe the process in more detail with the commands. n ArcGIS Pro, a shapefile of 50m buffers with all the attributes and columns of the original point‐data file was made. Dates were turned into integer values and were then split by the new integer‐value‐dates in into different shapefiles. The shapefiles were then iteratively pairwise dissolved—without creating multipart features—and all fields in the data except observation‐ID, shape, and coordinate‐related‐columns were considered as dissolve fields. Of the dissolved fields, e‐mail ID was the most relevant one. The “statistic field” was the field for observation‐ID, of which in case of overlap only the information of te first would be kept. The dissolved shapefiles were then appended into a single new shapefile. The new shapefile was spatially joined with the original point‐data, and only the points where the data of the original point‐data and the latter dissolved shapefile were the same—that is, all of the data within the points and buffers were identical—were selected and exported into a new shapefile. This shapefile was then exported into a CSV file. In Excel, the duplicate JOIN_FID’s would then be marked, and those that didn't have the same FIRST_id field as the original file's ID would be removed. This leaves you with the cleaned shapefile. In total, 85'465 of the original 86'863 observations of registered participants remained.

#### Procedure to remove duplicate observations among unregistered participants

For unregistered participants, the procedure was the same as for registered participants, with the alteration that any field related to email‐ID was not relevant. This made the cleaning of observations of the non‐registered participants somewhat more conservative, as overlapping observations on the same date could not be distinguished by participant if they had identical other metadata, and these would thus be considered indistinguishable and hence removed. In total, 18'922 of the original 20'577 observations of unregistered participants remained.

After both files were cleaned, they could again be merged to create the final cleaned dataset that was used for analyses. Finally, 104'387 of the original total of 107'440 observations remained.

### Other information available in the main LBV dataset

Many other types of information that were additionally collected in the Igel in Bayern platform were not used in this analysis, because they were not relevant to the question asked. This information included, but was not limited to, the living or dead status of the hedgehog, information on the habitat/place where the hedgehog was observed, and if potential death was caused by traffic. A full overview can be found on the website (https://www.igel‐in‐bayern.de/).

### Comparing observations across years

To compare the number of hedgehog observations in 2020 with those of 2016–2019, we used Generalized Additive Models. First, we smoothed weekly numbers of both data series (weeks 1–53 of 2020 and 2016–2019) separately with a thin plate spline function, using the “mgcv” package in R (Wood, 2021). Model smoothness was fitted using the generalized cross validation (GCV) option. Up to 53 knots were allowed for the smoothing function, to provide a detailed picture of changes in the number of hedgehog observations across the year and to not artificially limit the amount of wiggliness that CGV chooses. Model fit was assessed using the *R* ^2^ value and by visually assessing residuals. Simultaneous confidence intervals for penalized splines—based on the previously constructed models—were used to compare 2020 with 2016–2019 using the “itsadug” package, as they better reflect the uncertainty of the fitted functions than “across the function” confidence intervals (Van Rij et al., 2020). Briefly, confidence intervals are constructed in such a manner that approximately 95% of simulated draws from the posterior distribution fall withing the confidence interval. The R‐code for fitting the spline functions can be found in lines 73, 157, and 215 of the attached RMarkdown file. R‐code for comparing the fitted spline functions can be found in lines 88, 171, and 229 in the file Sweet_et_al_Code. Rmd uploaded with the manuscript.

### Assessment of urban/nonurban observations

Mean imperviousness density in the 200m radius surrounding each hedgehog observation was calculated in ArcGIS Pro using the “zonal statistics as table” tool. Groups of increments of 20% impervious surface density were then made in RStudio (see line 291–296 in the RMarkdown file). For impervious surface density data, the 20m*20m resolution 2015 Impervious Surface Density map of the European Union's Copernicus Land Monitoring Service (Langanke, 2016) was used.

95% prediction intervals on the percentage of observations and observers per imperviousness class, and measures of significance, were done in the same manner as the prediction models in Crimmins et al., 2021. Briefly, Crimmins et al. performed linear and polynomial models for each of their factor levels (in their case, the individual citizen science programs) for the years preceding 2020 and chose the best ones. Using these models, they created an expected 2020 value with a 95% prediction interval for 2020. They then compared the predicted 2020 value to the observed 2020 value, calculated the percent difference between the two, and assessed whether the observed value fell outside of the predicted 95% interval as a measure of significance.

Linear models were constructed for the time period 2016–2019, using the percentage of observations or observers that did observations within each imperviousness class in each year, and extrapolated into 2020 with the “predict” function in R (R Development Core Team, 2008) to create an expected value and 95% prediction interval for 2020 for the percentage of observations or observers within each imperviousness class. These predicted values for 2020 were then compared with the realized values for 2020, and if the realized value fell outside of the 95% prediction interval for 2020 this was treated as a significant deviation. This method has the benefit of accounting for ongoing changes throughout the preceding years—through the slope of the linear model from 2016–2019 for each imperviousness class—something that methods such as a chi‐square would not do. In the main text, only the comparisons between predicted and observed values for 2020 are shown. Here, the model outputs and the extrapolated values for 2020 will be shown. Figure A1 shows that for all the imperviousness classes the realized value for 2020 does not significantly deviate from the expected value, that is, there is no significant difference between the realized and expected values in the proportional number of observations in each imperviousness class in 2020. Figure A2 shows that only in the lowest imperviousness the realized number of observers significantly deviates from the expected number of observers. There is no significant difference between the realized and expected values in the proportional number of observers in the other classes in 2020.

R‐code for this analysis can be found from lines 288 onward in the attached RMarkdown file.
