# Supplementary material for: COVID‐19 lockdown measures impacted citizen science hedgehog observation numbers in Bavaria, Germany
Source: Ecol Evol. 2022 Jun 17;12(6):e8989. doi: 10.1002/ece3.8989 (PMC9204849; doi:10.1002/ece3.8989)
Supplement: Supplementary file 1 — AppendixS1 [file ECE3-12-0-s001.doc]

# Appendix

## A1 Duplicate observations

Because observations could be done by both registered and not-registered participants, of which only the observations of registered participants could be connected to an anonymized participant ID, while the non-registered participants by default got a Participant ID of '0', these two groups had to be cleaned individually.

For preparation, the data was split into two documents: one with registered participants and one with non-registered participants. After removal of duplicates, the final datasets were then again merged. Here we describe the steps to remove duplicate observations, followed by a reproducible ArcGIS-specific description.

Firstly, a shapefile (1) with 50m buffers around each datapoint was created. The shapefile with these buffers was then split into multiple shapefiles (2): one for each day in the dataset.

For each of these shapefiles, i.e. for every day of the calendar day where there were observations, the observation points were pairwise dissolved, so that two observations where the buffers overlapped to any extend and that had the same data on them would be considered as identical observations. If the observations were considered to be identical, i.e. referring to the same hedgehog seen by the same person, the two observation buffers would be merged, creating a larger buffer made from the combined dissolved buffers. These new buffers would gain the observation-ID of the first if the observations out of which the dissolved buffers were made.

After this was done for each created shapefile created in (2), i.e. for each day, these shapefiles (2) were merged to create a single shapefile with all the new dissolved buffers in it (3). This new shapefile was then spatially joined (overlapped) with the original point-data, and only points that had the same data as a buffer (from 3) that overlapped them were kept. This gave the data three different ID fields: (a) JOIN_FID’s, i.e. new ID’s made for points that were overlapped with a certain buffer; (b) FIRST_id’s, which were the ID’s of the first observation that was part of any combined buffer, and (c) the original observation-ID. These selected points were exported into a new shapefile, which was then exported into a CSV file. In Excel, the duplicate JOIN_FID’s would then be marked, and those that didn’t have the same FIRST_id field as the original file’s observation-ID would be removed. This was done for both the data with registered participants and with unregistered participants, albeit with some minor differences, as follows:

### a) Detailed ArcGIS procedure to remove duplicate observations among registered participants

Here we describe the process in more detail with the commands. n ArcGIS Pro, a shapefile of 50m buffers with all the attributes and columns of the original point-data file was made. Dates were turned into integer values and were then split by the new integer-value-dates in into different shapefiles. The shapefiles were then iteratively pairwise dissolved -without creating multipart features-, and all fields in the data except observation-ID, shape, and coordinate-related-columns were considered as dissolve fields. Of the dissolved fields, e-mail ID was the most relevant one. The “statistic field” was the field for observation-ID, of which in case of overlap only the information of te first would be kept. The dissolved shapefiles were then appended into a single new shapefile. The new shapefile was spatially joined with the original point-data, and only the points where the data of the original point-data and the latter dissolved shapefile were the same – i.e. all of the data within the points and buffers were identical – were selected and exported into a new shapefile. This shapefile was then exported into a CSV file. In Excel, the duplicate JOIN_FID’s would then be marked, and those that didn’t have the same FIRST_id field as the original file’s ID would be removed. This leaves you with the cleaned shapefile. In total, 85'465 of the original 86'863 observations of registered participants remained.

### b) Procedure to remove duplicate observations among unregistered participants

For unregistered participants, the procedure was the same as for registered participants, with the alteration that any field related to email-ID was not relevant. This made the cleaning of observations of the non-registered participants somewhat more conservative, as overlapping observations on the same date could not be distinguished by participant if they had identical other metadata, and these would thus be considered indistinguishable and hence removed. In total, 18'922 of the original 20'577 observations of unregistered participants remained.

After both files were cleaned, they could again be merged to create the final cleaned dataset that was used for analyses. Finally, 104'387 of the original total of 107'440 observations remained.

## A2 Other information available in the main LBV dataset

Many other types of information that were additionally collected in the Igel in Bayern platform were not used in this analysis, because they were not relevant to the question asked. This information included, but was not limited to, the living or dead status of the hedgehog, information on the habitat/place where the hedgehog was observed, and if potential death was caused by traffic. A full overview can be found on the website (https://www.igel-in-bayern.de/).

## B. Comparing observations across years

To compare the number of hedgehog observations in 2020 with those of 2016-2019, we used Generalized Additive Models. First, we smoothed weekly numbers of both data series (weeks 1-53 of 2020 and 2016-2019) separately with a thin plate spline function, using the ‘mgcv’ package in R (Wood, 2021). Model smoothness was fitted using the generalized cross validation (GCV) option. Up to 53 knots were allowed for the smoothing function, to provide a detailed picture of changes in the number of hedgehog observations across the year and to not artificially limit the amount of wiggliness that CGV chooses. Model fit was assessed using the R^2^ value and by visually assessing residuals. Simultaneous confidence intervals for penalized splines – based on the previously constructed models – were used to compare 2020 with 2016-2019 using the ‘itsadug’ package, as they better reflect the uncertainty of the fitted functions than ‘across the function’ confidence intervals (Van Rij et al., 2020). Briefly, confidence intervals are constructed in such a manner that approximately 95% of simulated draws from the posterior distribution fall withing the confidence interval. The R-code for fitting the spline functions can be found in lines 73, 157 and 215 of the attached RMarkdown file. R-code for comparing the fitted spline functions can be found in lines 88, 171, and 229 in the file Sweet_et_al_Code.Rmd uploaded with the manuscript.

## C. Assessment of urban/non-urban observations

Mean imperviousness density in the 200m radius surrounding each hedgehog observation was calculated in ArcGIS Pro using the ‘zonal statistics as table’ tool. Groups of increments of 20% impervious surface density were then made in RStudio (see line 291-296 in the RMarkdown file). For impervious surface density data, the 20m*20m resolution 2015 Impervious Surface Density map of the European Union’s Copernicus Land Monitoring Service (Langanke, 2016) was used.

95% prediction intervals on the percentage of observations and observers per imperviousness class, and measures of significance, were done in the same manner as the prediction models in Crimmins et al., 2021. Briefly, Crimmins et al. performed linear and polynomial models for each of their factor levels (in their case, the individual citizen science programs) for the years preceding 2020 and chose the best ones. Using these models, they created an expected 2020 value with a 95% prediction interval for 2020. They then compared the predicted 2020 value to the observed 2020 value, calculated the percent difference between the two, and assessed whether the observed value fell outside of the predicted 95% interval as a measure of significance.

Linear models were constructed for the time period 2016-2019, using the percentage of observations or observers that did observations within each imperviousness class in each year, and extrapolated into 2020 with the ‘predict’ function in R (R Development Core Team, 2008) to create an expected value and 95% prediction interval for 2020 for the percentage of observations or observers within each imperviousness class. These predicted values for 2020 were then compared with the realized values for 2020, and if the realized value fell outside of the 95% prediction interval for 2020 this was treated as a significant deviation. This method has the benefit of accounting for ongoing changes throughout the preceding years – through the slope of the linear model from 2016-2019 for each imperviousness class –, something that methods such as a chi-square would not do. In the main text, only the comparisons between predicted and observed values for 2020 are shown. Here, the model outputs and the extrapolated values for 2020 will be shown. Fig. A1 shows that for all the imperviousness classes the realized value for 2020 does not significantly deviate from the expected value, i.e. there is no significant difference between the realized and expected values in the proportional number of observations in each imperviousness class in 2020. Fig. A2 shows that only in the lowest imperviousness the realized number of observers significantly deviates from the expected number of observers. There is no significant difference between the realized and expected values in the proportional number of observers in the other classes in 2020.

R-code for this analysis can be found from lines 288 onward in the attached RMarkdown file.


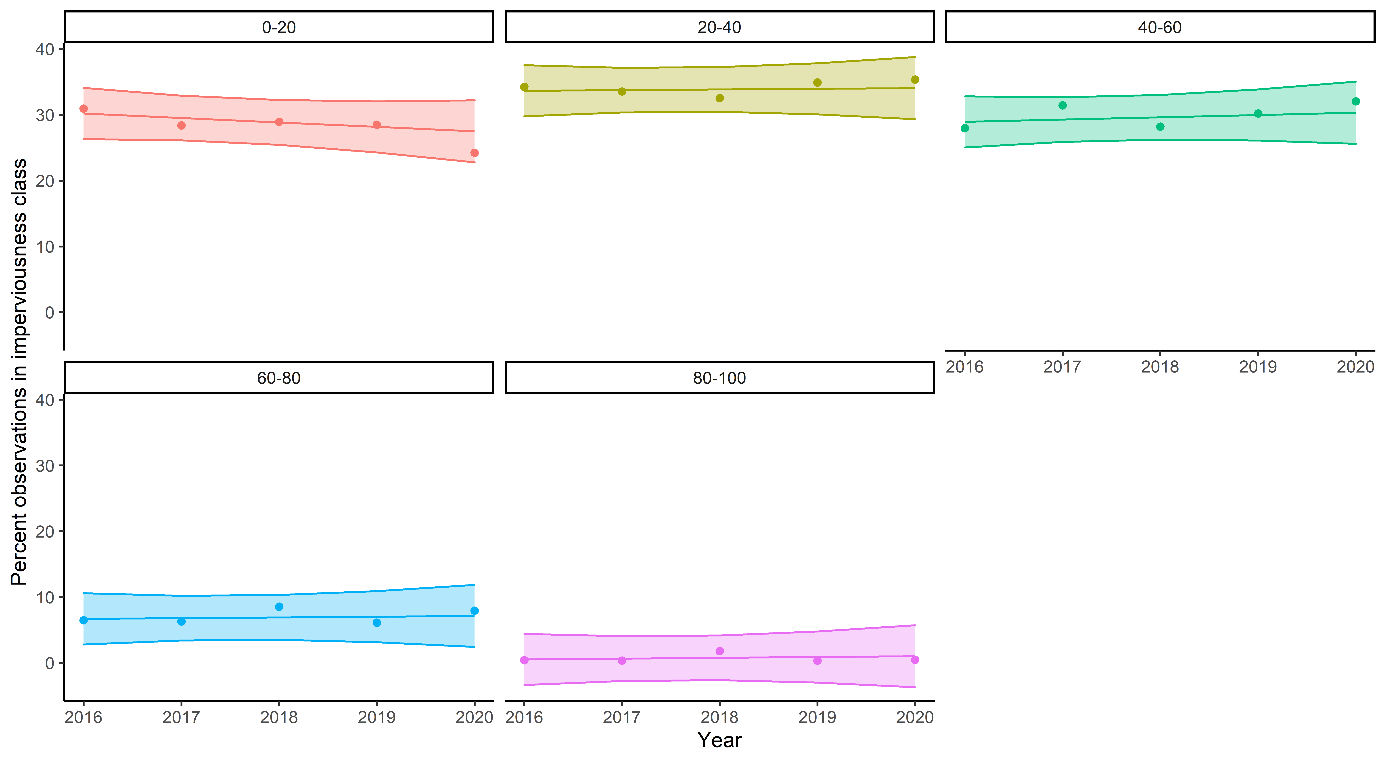


Figure A1. Percentage of observations within urbanization classes from 2016-2019, extrapolated into 2020. Each panel shows the results for one imperviousness classes. The dots indicate the yearly realized percentage of observation in each imperviousness class each year. In none of the imperviousness classes, the realized value of 2020 significantly differed from extrapolated expectations. Y-axis: The percent of observations in each imperviousness class. X-axis: Year. Plot facets and colours: Imperviousness classes. Line ± CI: Linear model per imperviousness class based on 2016-2019, extrapolated towards 2020.


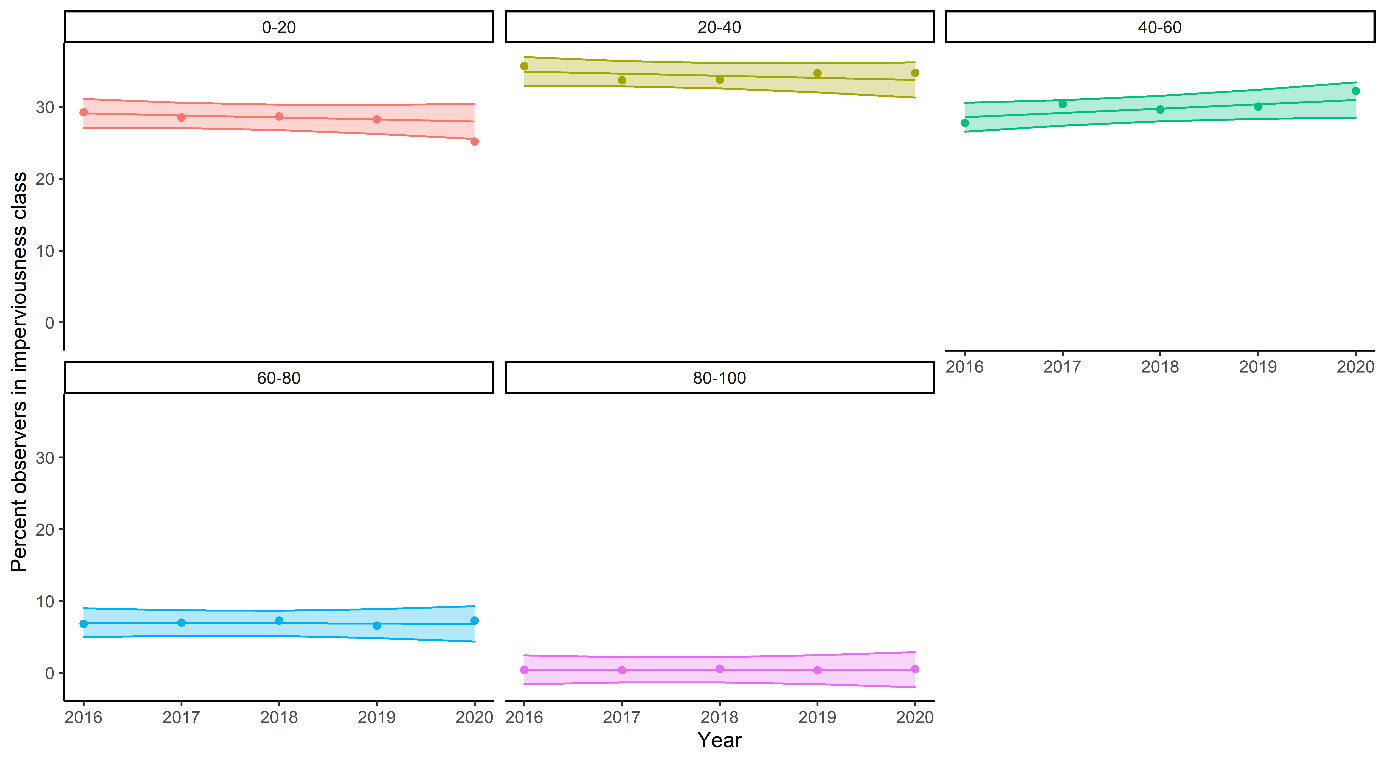


Figure A2. Percentage of participants (observers) within urbanization class from 2019-2019, extrapolated into 2020. Each panel shows the results for one imperviousness classes. The dots indicate the yearly realized percentage of observers in each imperviousness class each year. There is a very mild deviation in realized value from expected value in the lowest imperviousness class. No significant deviations in the other imperviousness classes. Y-axis: The percent of observers in each imperviousness class. X-axis: Year. Plot facets and colours: Imperviousness classes. Line ± CI: Linear model per imperviousness class based on 2016-2019, extrapolated towards 2020.

# Appendix References

Crimmins, T. M., Posthumus, E., Schaffer, S., & Prudic, K. L. (2021). COVID-19 impacts on participation in large scale biodiversity-themed community science projects in the United States. *Biological Conservation*, *256*, 109017. https://doi.org/10.1016/j.biocon.2021.109017

Langanke, T. (2016). *Copernicus Land Monitoring Service – High Resolution Layer Imperviousness: Product Specifications Document*. Copernicus team at EEA. https://land.copernicus.eu/user-corner/technical-library/hrl-imperviousness-technical-document-prod-2015

R Development Core Team. (2008). *R: A language and environment for statistical computing.* R Foundation for Statistical Computing. http://www.r-project.org.

Van Rij, J., Wieling, M., Baayen, R. H., & van Rijn, H. (2020). *itsadug: Interpreting Time Series and Autocorrelated Data Using GAMMs* (2.4) [Computer software].

Wood, S. N. (2021). *Mixed GAM Computation Vehicle with Automatic Smoothness Estimation*. https://cran.r-project.org/package=mgcv
